# Frequency of Participation in a Return-to-Work Program Predicts Successful Work Restoration but Not Maintenance

**DOI:** 10.3389/fpsyt.2021.620520

**Published:** 2021-05-14

**Authors:** Yuriko Hoaki, Takeshi Terao

**Affiliations:** ^1^Department of Psychiatry, Oita Okanoue Hospital, Oita, Japan; ^2^Department of Neuropsychiatry, Oita University Faculty of Medicine, Oita, Japan

**Keywords:** intervention, restoration, sick leave, worker, mood disorder, anxiety disorder, return-to-work program

## Abstract

Several predictors for work restoration and maintenance of restoration have been examined among employees with mood and/or anxiety disorders, but whether frequency of participation in a return-to-work (RTW) program can predict successful work restoration and maintenance remains unclear. In the present study, we attempted to investigate the hypothesis that the frequency of RTW program participation can predict successful work restoration and maintenance. Among seventy-seven patients attending an RTW program, the frequency of participation was positively associated with work restoration but not with maintenance. The result was in partial agreement (restoration) and partial disagreement (maintenance of restoration) with our hypothesis. Thus, the present study suggests that the frequency of participation in an RTW program can predict successful work restoration but not maintenance.

## Introduction

Several predictors of work restoration among employees with mood and/or anxiety disorders have been examined by past studies. With respect to work participation, Lagerveld et al. (1) performed a systematic review focusing on depression and found strong evidence for the association between a long duration of depressive episodes and work disability. Moderate evidence was found for the associations of work disability with more severe types of depressive disorder, presence of comorbid mental or physical disorders, older age, and a history of previous sick leave. With respect to work functioning, severe depressive symptoms were associated with work limitations, and clinical improvement was related to work productivity (moderate evidence) (1). In another systematic review, Cornelius et al. (2) found strong evidence for the association of older age (>50 years) with continuing disability and longer time to return to work (RTW); limited evidence for the association of other personal factors (gender, education, history of previous sickness absence, negative recovery expectation, socio-economic status), health-related factors (stress-related and shoulder/back pain, depression/anxiety disorders), and job-related factors (unemployment, quality and continuity of occupational care, supervisor behavior) with disability and RTW; and limited evidence for the association of personal/external factors (education, sole breadwinner, partial/full RTW, changing work tasks) with symptom recovery.

Regarding interventions that may enhance RTW, systematic reviews suggest that medication, enhanced primary care, and psychotherapeutic interventions aimed at symptom reduction [e.g., cognitive behavioral therapy (CBT)] do not improve RTW among employees on sick leave due to mental health problems (3, 4). In contrast, psychotherapeutic interventions that included a work-focused component showed encouraging results (5–7). While work-focused interventions differ with studies and generally consist of multiple components (8, 9), successful interventions appear to combine an early, gradual RTW with work-focused CBT and/or problem-solving therapy (9). Gradual RTW means that employees resume their work step-by-step in terms of work hours and tasks until they have fully returned to work. Interestingly, interventions that were effective in terms of RTW did not result in larger reductions in psychological complaints compared with control groups (5, 6). Despite some promising findings, interventions with a work-focused component did not always enhance RTW (9–12). A recent meta-analysis (13) suggests that work-directed interventions (occupational therapy and multi-component work intervention incorporating work modification and support) combined with a clinical intervention can be effective in reducing sickness absence and that enhancing occupational or primary care with CBT and structured telephone outreach with care management that includes medication has the potential to reduce sick leave; however, the number of related studies is small. Salomonsson et al. (14) performed a randomized trial that compared the effects of CBT, RTW intervention, and a combination of them (COMBO) on a stress subgroup (*n* = 152 with adjustment disorder or exhaustion disorder) and DepAnxIn subgroup (*n* = 59 with depression, anxiety disorders, or insomnia). The stress subgroup showed a superior reduction of stress-related symptoms after CBT compared to RTW intervention, but there was no significant difference between the COMBO and RTW intervention or CBT. Moreover, there was no difference between treatments with respect to days on sick leave the year after randomization. In the DepAnxIn group, there was no significant difference between treatments with respect to symptom reduction. With respect to days on sick leave, patients had 92 fewer days on sick leave after RTW intervention compared to CBT and 76 fewer days on sick leave after COMBO compared to CBT. Thus, RTW intervention may be more effective than CBT for the DepAnxIn subgroup rather than the stress subgroup, but RTW intervention often includes CBT, and it seems difficult to identify the specific effects of RTW intervention. In addition, sick leave days are important because it reflects directly mental and physical health and indirectly work stress, which is linked to the effects of RTW intervention.

To date, it has not been investigated whether the frequency of participation in an RTW program is associated with successful work restoration and maintenance of restoration. It seems plausible that more frequent RTW program participation would entail more success in work restoration and maintenance. On the other hand, the duration of participation in an RTW program might be affected arbitrarily by the circumstances of individual companies to which each patient would be restored. For example, some workers may take relatively longer sick leave days whereas other workers can take relatively shorter sick leave days due to the rules of individual companies. Rather, the frequency is, to some extent, free from the circumstances of individual companies because for example one can try to participate in an RTW program 5 days per week for 4 weeks which is equal to a participation of 2 days per week for 10 weeks in the frequency of 20 if the upper limitation of sick leave days is 4 weeks. Thus, in the present study, we attempted to investigate the hypothesis that the frequency of RTW program participation is positively associated with successful work restoration and maintenance.

## Materials and Methods

### Subjects

Oita Okanoue Hospital in Japan had a rehabilitation unit that conducted the RTW program. Strictly speaking, there were two programs—old and new. The old program was in effect from November 2013 to May 2017, and the new program has been implemented since June 2017. The last patient of this study started the new program on February 10, 2020, and this patient and other patients were followed up to September 1, 2020. The patients with new program were followed up for 577.4 ± 302.2 days (range: 62 to 1,096 days). The old program had entry criteria of Beck Depression Inventory scores equal to or <12 and capability of pre-participation in pre-RTW program 3 times a week for 2 weeks, while the new one had entry criteria of Hamilton Depression Rating Scale scores equal to or <9 and capability of pre-participation in pre-RTW program 3 times a week for 2 weeks. The schedule of the old program included office work, meeting, yoga, CBT, social skill training, and light sports, while that of the new program included an additional element of cognitive training such as operating a personal computer for composition, reading, calculation, and so on. Of the 77 participants (56 men and 21 women) in this study, 44 patients had attended the old program and 33 patients attended the new program. Their mean age was 43.2 ± 8.5 (standard deviation) years. The psychiatric diagnoses according to the International Classification of Diseases Tenth Revision were F31 (*N* = 9), F32 (*N* = 38), F34 (*N* = 1), F40 (*N* = 3), F41 (*N* = 1), F42 (*N* = 2), F43 (*N* = 21), F44 (*N* = 1), and F45 (*N* = 1). That is, there were 48 patients with mood disorders and 29 patients with anxiety disorders. The mean total work experience was 21.7 ± 9.7 years. The frequency of RTW program participation was 67.8 ± 41.2 times. Informed consent was obtained from 33 patients attending the new program, which was approved by the ethics committee of Oita University Faculty of Medicine, and the alternative opt-out method was applied to 44 patients attending the old program, which was approved by the ethics committee of Oita Okanoue Hospital.

We investigated whether these patients could be restored to their jobs, and after restoration, whether they could maintain the restoration, and we attempted to investigate the associations of work restoration and maintenance with the frequency of RTW program participation. Maintenance of restoration was defined as the duration (days) from restoration to the next leave of absence from work or to the last observation while keeping restoration.

### Statistical Analyses

The association between the frequency of RTW program participation and work restoration was compared between patients who could be restored to their jobs (restoration group) and those who could not be restored (non-restoration group) using a *t*-test (crude model). Next, a binary logistic regression analysis was performed using restoration or non-restoration as a dependent factor, and age, gender, frequency of RTW program participation, program (old or new), and total work experience (years) as independent factors (adjusted model).

The association of RTW program participation with maintenance of work restoration was analyzed using the Kaplan-Meier survival analysis (crude model) and Cox regression analysis, wherein whether restoration was maintained or not was the dependent factor and age, gender, frequency of RTW program participation, program (old or new), and total work experience (years) were independent factors (adjusted model). SPSS Statistics 26 was used for these analyses.

## Results

The restoration group had a significantly higher frequency of RTW program participation than the non-restoration group (72.1 ± 42.1 vs. 44.0 ± 26.1 times; *t* = 3.1, *p* = 0.005) (Table 1). The range of the duration of RTW participation was 8 to 318 days and the mean was 134.1 days and SD was 67.4 days. The binary logistic regression analysis with forced entry showed the significance of the model (χ^2^ = 12.594, *p* = 0.027) and that the frequency of RTW program participation was significantly associated with restoration (more frequency, more restoration), but not with the other factors (Table 1). Particularly, whether the RTW program was old or new was not associated with the outcome. In addition, we performed another binary logistic regression analysis with forward stepwise selection method to decrease the number of independent factors for only 77 patients, showing the significance of the model (χ^2^ = 9.915, *p* = 0.007) with only two independent factors (the frequency of RTW program participation and total work experience) and that the frequency of RTW program participation was significantly associated with restoration (odds ratio = 0.977, *p* = 0.021; more frequency, more restoration), but not with total work experience (odds ratio = 1.084, *p* = 0.062). This result was in agreement with that of forced entry with five independent factors.

**Table 1:** Results of t or χ^2^-test and binary logistic regression analysis of restoration and non-restoration groups.

	**Restoration**	**Non-restoration**	***t* or χ^**2**^-values**	**Odds ratio**	**95% Confidence interval**		***p***
	**Group (*N* = 65)**	**Group (*N* = 12)**	***p***		**Lower limit**	**Upper limit**	
Age (years)	42.7 ± 8.7	45.8 ± 7.0	*t* = −1.13, *p* = 0.261	0.876	0.708	1.083	0.220
Gender (female: male)	17: 48	4: 8	χ^2^ = 0.263, *p* = 0.608	1.278	0.253	6.446	0.766
Frequency of RTW program participation	72.1 ± 42.1	44.0 ± 26.1	*t* = 3.1, *p* = 0.005	0.973	0.952	0.995	0.016
Program (old: new)	36: 29	8: 4	χ^2^ = 0.526, *p* = 0.465	0.464	0.097	2.216	0.336
Total work experience (years)	20.8 ± 9.8	26.3 ± 8.3	*t* = −1.8, *p* = 0.076	1.194	0.986	1.448	0.070

*RTW, return-to-work*.

*Binary logistic regression analysis with forced entry showed the significance of the model (χ^2^ = 12.594, p = 0.027) and that the frequency of RTW program participation was significantly associated with work restoration (more frequency, more restoration), but not with the other factors*.

**Figure 1:**
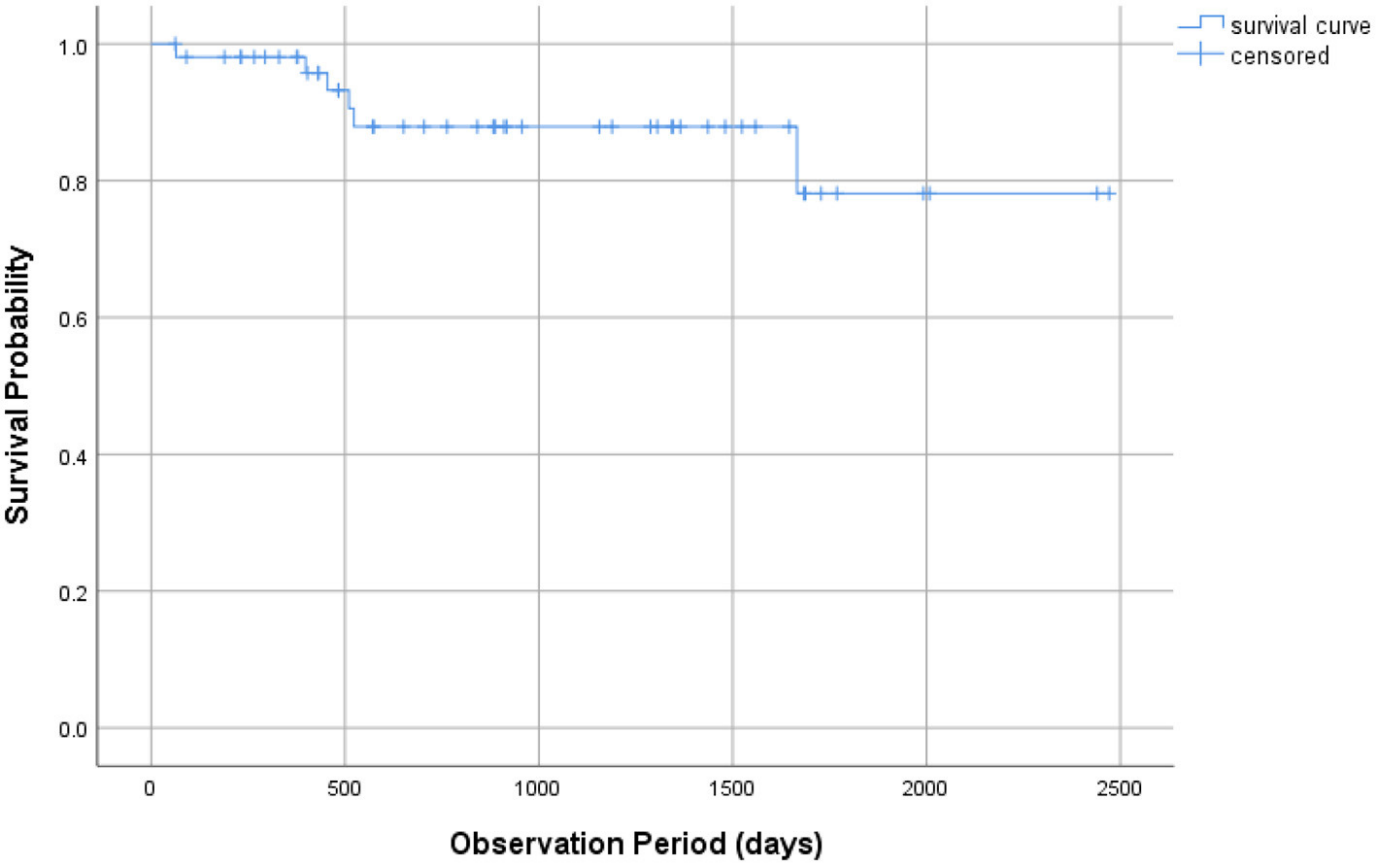
Kaplan–Meier survival analysis.

**Figure 2:**
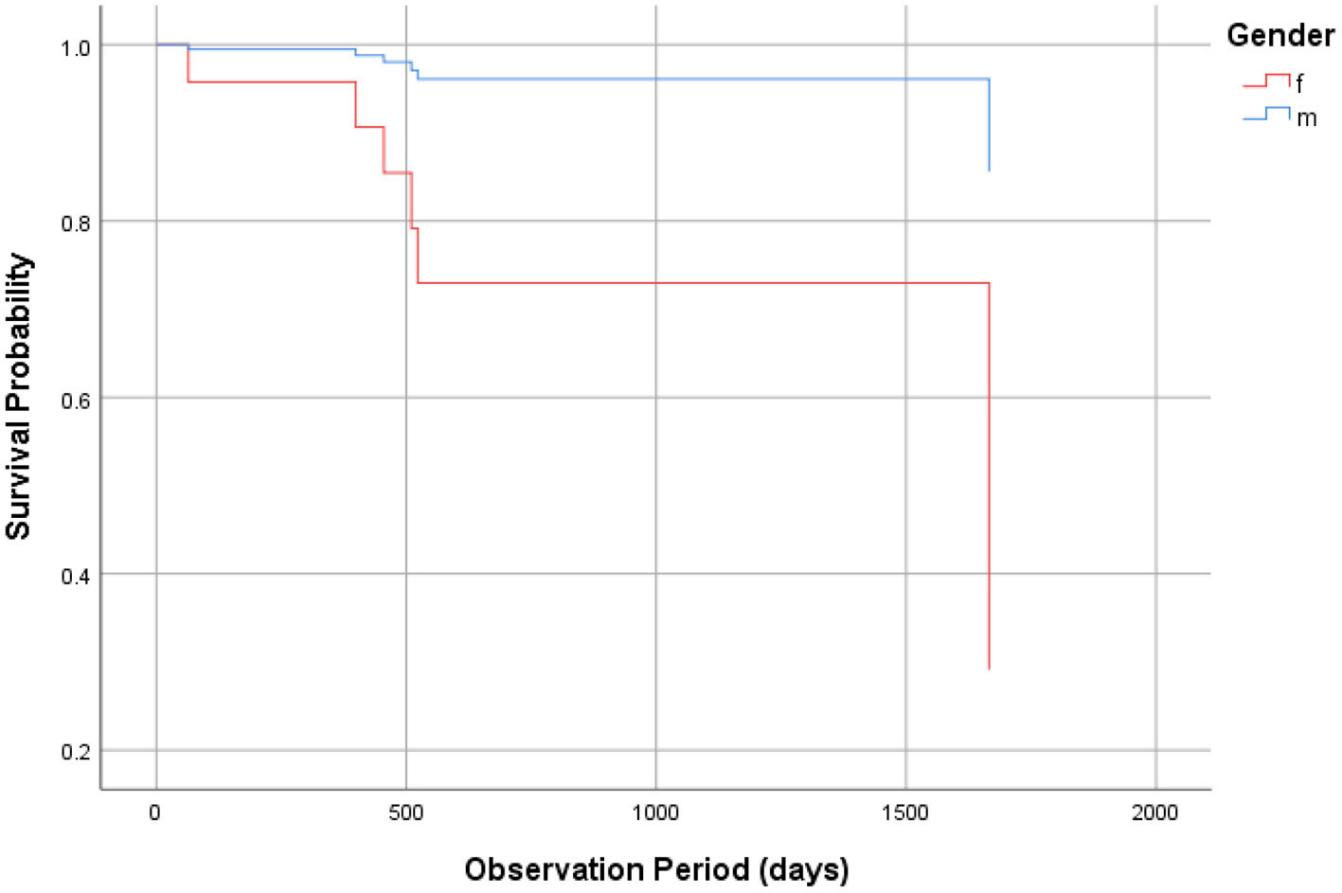
Cox regression analysis.

The Kaplan-Meier survival analysis showed the survival curve (Figure 1). The mean estimated survival time was 2142.8 ± 124.9 days. The Cox regression analysis with stepwise backward elimination (Wald) showed the significance of the model (χ^2^ = 7.225, *p* = 0.027) and that maintenance of restoration was significantly associated with gender (significantly better among men than women, Table 2, Figure 2) but not with the frequency of RTW program participation (Table 2).

**Table 2:** Cox regression analysis of maintaining restoration.

	**Odds ratio**	**95% Confidence interval**		***P***
		**Lower limit**	**Upper limit**	
Gender	7.928	1.329	47.300	0.023
Frequency of RTW program participation	1.027	0.995	1.059	0.096

*RTW, return-to-work*.

*Cox regression analysis with stepwise backward elimination (Wald) showed the significance of the model (χ^2^ = 7.225, p = 0.027) and that maintenance of restoration was significantly associated with gender (significantly better among men than women), but not with the frequency of RTW program participation*.

## Discussion

The present findings show that the frequency of RTW program participation is positively associated with work restoration but not with maintenance of restoration. This is in partial agreement (restoration) and partial disagreement (maintenance of restoration) with our hypothesis. It is possible that the effects of the RTW program disappeared over time. Interestingly, maintenance of restoration was significantly associated with gender (significantly better among men than women). It seems possible that when confronting difficulties in maintaining work restoration, female workers may be prone to discontinuing their jobs than male workers, but this should be clarified in future research.

Several reports have shown predictive factors for maintenance of restoration. Hori et al. (15) reported that worse social adaptation self-evaluation scale (SASS) scores, worse 3-back correct response rate, and more benzodiazepine dosage were associated with further episodes of sick leave. Atake et al. (16) reported that successful restoration (with a year of restoration maintenance) was predicted by an SASS score more than or equal to 31, a correct response rate more than or equal to 50% in the 3-back task, and a diazepam equivalent benzodiazepine dosage ≤ 7.5 mg at the time of deciding on restoration. In addition, Morita et al. (17) showed that <2 h of activity outside of the home predicted further sick leave. Further studies should investigate whether various such predictors can correctly predict maintenance of restoration.

Sproken et al. (18) identified five distinct RTW trajectories, namely (a) fast RTW with little chance of relapse, (b) slow RTW with little chance of relapse, (c) fast RTW with considerable chance of relapse, (d) slow RTW with considerable chance of relapse, and (e) very fast RTW with a very small chance of relapse. Stress complaints and adjustment disorders were more prevalent in the faster trajectories, while depression and burnout were more prevalent in the slower trajectories. Furthermore, older employees, women, and non-profit sector employees showed longer trajectories. Although our study did not pursue such trajectories, our findings of less restoration in female workers may be in line with their findings of faster restoration dampening female restoration requiring slower-pace restoration.

The limitations of this study are the relatively small sample size and that the RTW location was a private hospital, which might preclude generalization. Moreover, there were some other confounding factors that might be associated with work restoration and maintenance, such as differences in psychiatric diagnosis and severity of the disorders. Owing to the nature of a retrospective observational study, it was difficult to clarify the causal relationship between RTW program and work restoration and maintenance. Although the RTW program in this hospital seems to be average in Japan, it cannot be denied that the RTW program and/or the participants and/or the staffs were deviated and the present findings were also deviated. Moreover, various workers from several companies participated in the RTW program which provided various tasks for several duration. That is, the present findings derived from heterogenous situation. Moreover, honestly speaking, it is unknown whether the present findings are reconfirmed or not and it cannot be completely denied that there is a bell-shaped dose relationship where optimal frequency exists and less or more than the optimal frequency is not good for restoration. Further studies are required to resolve these problems.

In conclusion, the present study suggests that the frequency of RTW program participation can predict successful work restoration but not maintenance of restoration.

## Data Availability Statement

The raw data supporting the conclusions of this article will be made available by the authors, without undue reservation.

## Ethics Statement

Informed consent was obtained from 33 patients attending the new program, which was approved by the ethics committee of Oita University Faculty of Medicine, and the alternative opt-out method was applied to 44 patients attending the old program, which was approved by the ethics committee of Oita Okanoue Hospital.

## Author Contributions

YH and TT planned this study, analyzed the data, discussed the results, wrote the manuscript, and approved the final manuscript. YH collected the data.

## Conflict of Interest

The authors declare that the research was conducted in the absence of any commercial or financial relationships that could be construed as a potential conflict of interest.
